# Outcome-driven dosimetry optimization for [^177^Lu]Lu-PSMA-617 radiopharmaceutical therapy: proof of concept on single time point dosimetry optimization

**DOI:** 10.1007/s00259-026-07936-w

**Published:** 2026-06-13

**Authors:** Jiaxi Hu, Robert Seifert, Carlos Vinicius Gomes, Yizhou Chen, Yibin Liu, Warissara Jutidamrongphan, Michelle Amon, Jiahui Wang, Song Xue, Axel Rominger, Ali Afshar-Ormieh, Kuangyu Shi

**Affiliations:** 1 Department of Nuclear Medicine, Inselspital, Bern University Hospital, University of Bern, Freiburgstrasse 20 3010 Bern, Bern, Switzerland; 2 Biomedical Imaging Laboratory (BIG), Department of Electrical and Computer Engineering, Faculty of Science and Technology, University of Macau, Taipa, Macau SAR China; 3 Graduate School for Health Sciences, University of Bern, Bern, Switzerland; 4 Department of Biomedical Imaging and Image-Guided Therapy, Division of Nuclear Medicine, Medical University of Vienna, Vienna, Austria

**Keywords:** *[*^*177*^*Lu]Lu-PSMA-617*, *Dosimetry*, *Prostate cancer*, *Theranostics*, *Radiopharmaceutical therapy*

## Abstract

**Purpose:**

Internal dosimetry in radiopharmaceutical therapy (RPT) traditionally prioritizes methodological optimization driven by physical dose accuracy. However, even recommended multiple-time-point (MTP) dosimetry remains subject to uncertainties related to limited sampling schedules, pharmacokinetic, and curve-fitting modeling assumptions. In patients with metastatic castration-resistant prostate cancer (mCRPC) treated with [^177^Lu]Lu-PSMA-617 RPT, we explored an outcome-driven dosimetry optimization strategy by comparing MTP and single-time-point (STP) dosimetry, and identifying optimal time-points (TPs) for Hänscheid approximation based on therapy outcomes.

**Methods:**

Clinical and image data from 50 patients were retrospectively analyzed. Transient treatment-emergent adverse events (TEAEs) and prostate-specific antigen (PSA) response were monitored following CTCAE v5.0 and PCWG3. Cycle-level mean absorbed doses (*MTPD*_*tox*_, and *1d-*, *2d-*, *3d-STPD*_*tox*_ based on single SPECT acquisitions at 1-, 2-, 3-day(s) p.i.) were computed for organs-at-risk and whole-body tumors. Additionally, cumulative absorbed doses (*STPD*_*cum*_ & *MTPD*_*cum*_) were derived for whole-body tumors.

**Results:**

Bone marrow STP dosimetry correlated significantly with anaemia grading (*1d-STPD*_*tox*_: Spearman’s ρ=0.35;*2d-STPD*_*tox*_: Spearman’s ρ=0.41; *3d-STPD*_*tox*_: Spearman’s ρ=0.46; all *p*_*adj*_<0.001), aligning with *MTPD*_*tox*_ (Spearman’s ρ=0.43, *p*<0.001). Williams’ *F*-test confirmed no significant difference in correlation strength between *STPD*_*tox*_ & *MTPD*_*tox*_ derived correlations at any TPs. For PSA response, both *MTPD*_*cum*_ (Spearman’s ρ=-0.26, *p*_*adj*_<0.05) and *STPD*_*cum*_ (*1d-*&*2d-STPD*_*cum*_: Spearman’s ρ=-0.34, *p*_*adj*_<0.001) showed significant correlations, without statistically significant differences between *STPD*_*cum*_ & *MTPD*_*cum*_ derived correlations.

**Conclusion:**

The outcome-driven TP selection for Hänscheid-based STP dosimetry converges with the physics-based choice within 2-day p.i., the clinically driven approach offers an alternative and complementary strategy for dosimetry development and may facilitate its translation into clinical practice.

**Supplementary Information:**

The online version contains supplementary material available at https://doi.org/10.1007/s00259-026-07936-w.

## Introduction

Dosimetry plays a key role in radiopharmaceutical therapy (RPT) by enabling individualized assessment of radiation exposure to target lesions and organs at risk (OARs). While dosimetry has traditionally been treated as a physical concept and has advanced through improvements towards physical accuracy, includes optimized quantitative SPECT reconstruction [1–3], curve-fitting method development [4], pharmacokinetic modeling innovations [5, 6], improved image processing [7, 8], and advanced dosimetry calculation methods incorporating radiobiological assumptions, Monte Carlo simulations, and deep learning, which have further refined voxel-level dose assessments [9–11]. In clinical practice, however, the primary purpose of dosimetry is not methodological perfection but the optimization of treatment outcomes. The European Council Directive 2013/59/EURATOM requires that exposures of target volumes be individually planned and verified, while non-target tissues are protected [12]. This regulatory framework highlights the importance of individualized dosimetry in RPT, and motivates a shift from purely physical dose accuracy toward therapy outcome-oriented dosimetry to better support patient-specific management [13].

The comprehensive multiple-time-point (MTP) dosimetry approach is commonly used as a reference framework [14]. However, its applicability is limited by the substantial logistical burden associated with repeated imaging, patient compliance, and resource-intensive data processing. Additionally, standardized and robust fittable pharmacokinetic models applicable across different patients, organs, or radiopharmaceuticals remain lacking [15–18], which can introduce inaccuracies due to limited sampling, reliance on oversimplified population-based assumptions, potentially hampering methodology development and reducing clinical applicability [19]. In response, simplified dosimetry approaches aim to improve the clinical feasibility of dosimetry by reducing the number of acquisition time-points (TPs) [5, 20–25], or by employing simulation-based [26, 27] or AI-assisted algorithms for streamlined dosimetry predictions [28, 29]. Single-time-point (STP) dosimetry has shown acceptable physical accuracy in several studies, but remains sensitive to the selection of imaging TPs and may introduce biased dose estimates compared to MTP dosimetry [23, 25, 30–32]. Beyond methodological considerations, studies have demonstrated clinically meaningful associations between dosimetry-derived doses and treatment outcomes. For example, tumor absorbed doses have been shown to differ significantly among PSA response groups [33–35], and Grob et al. further validated that a 2-TPs approach can effectively monitor hematologic safety [24]. Despite these advances, there is still no consensus on how to identify the optimal TP for STP dosimetry to maximize clinical utility. Existing approaches primarily emphasize methodological accuracy, while integrating treatment outcome information into dosimetry protocol design remains limited, underscoring the need to explore outcome-driven strategies to optimize simplified dosimetry workflows.

**Fig. 1:**
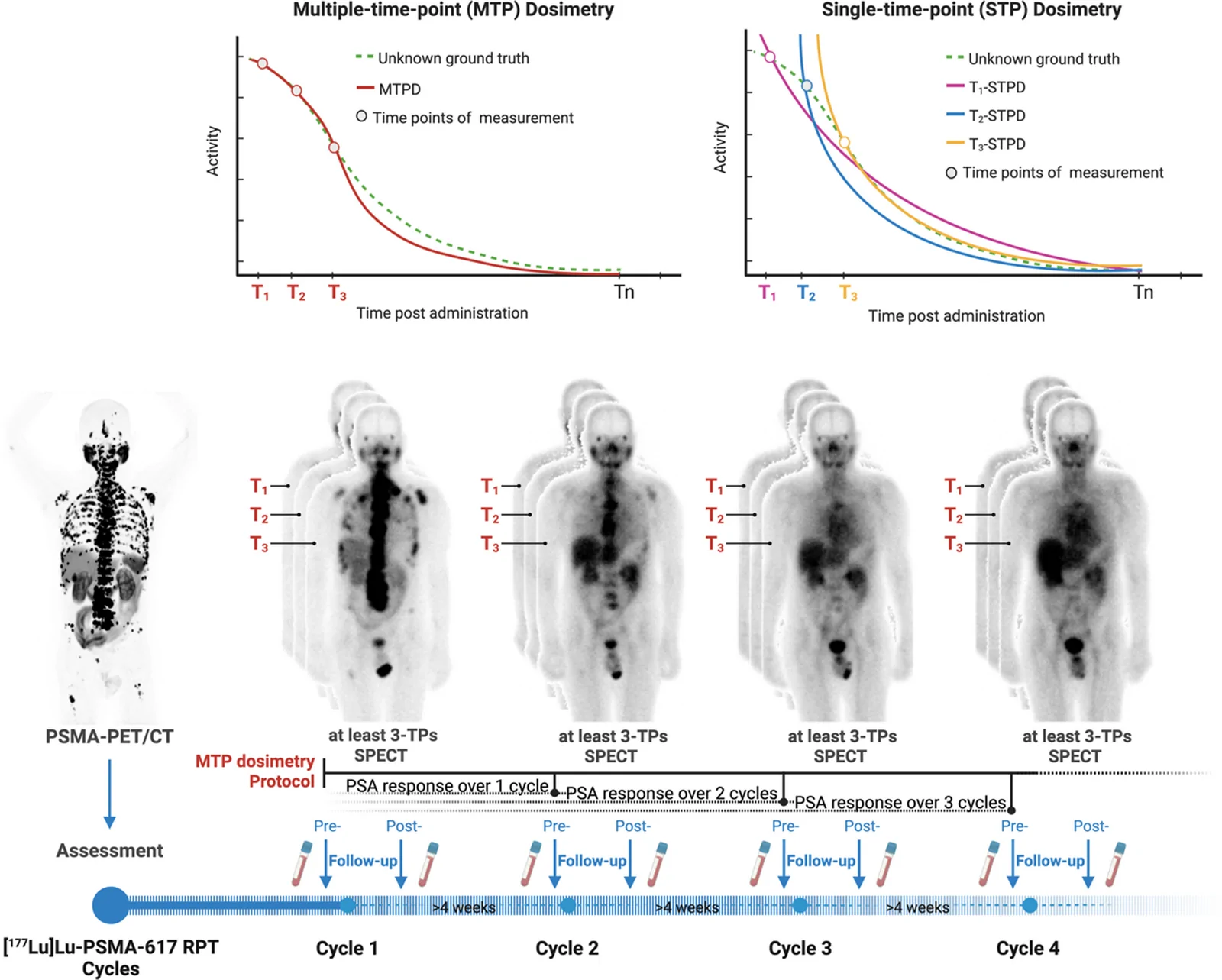
Study Overview. Pre-therapeutic PSMA-PET/CT study demonstrated intense PSMA uptake in multiple osseous and nodal metastases in a sample patient. Post-therapeutic quantitative SPECT/CT scans were acquired after each cycle of [^177^Lu]Lu-PSMA-617 RPT administration. For MTP dosimetry estimation, at least three time points (e.g., T_1_, T_2_, T_3_) were used to generate time-activity curves. In parallel, each available single time point (e.g., T_1_ p.i.) was analyzed for STP dosimetry (referred to as T_1_-STPD)

This study focused on [^177^Lu]Lu-PSMA-617 RPT for metastatic castration-resistant prostate cancer (mCRPC), which has demonstrated significant efficacy and survival benefits [36, 37]. However, approximately one-third of patients fail to receive optimal therapy outcomes, often due to inter-patient variability and the absence of personalized, outcome-driven monitoring strategies [38–40]. Our previous work found that STP dosimetry demonstrates significant correlations with therapy outcomes comparable to those observed with MTP dosimetry [41]. However, the optimal TP identification for STP dosimetry, particularly with respect to its ability to correlate STP-driven absorbed dose estimates with therapy outcomes, remains underexplored (shown in Figure 1). Therefore, this study aimed to investigate a proof-of-concept approach to outcome-driven dosimetry optimization by evaluating whether absorbed dose estimates derived from optimally timed STP imaging can effectively correlate with treatment outcomes, thereby directly linking the absorbed dose calculated via optimal STP to treatment outcomes.

## Materials and methods

### Patients characteristics

**Fig. 2:**
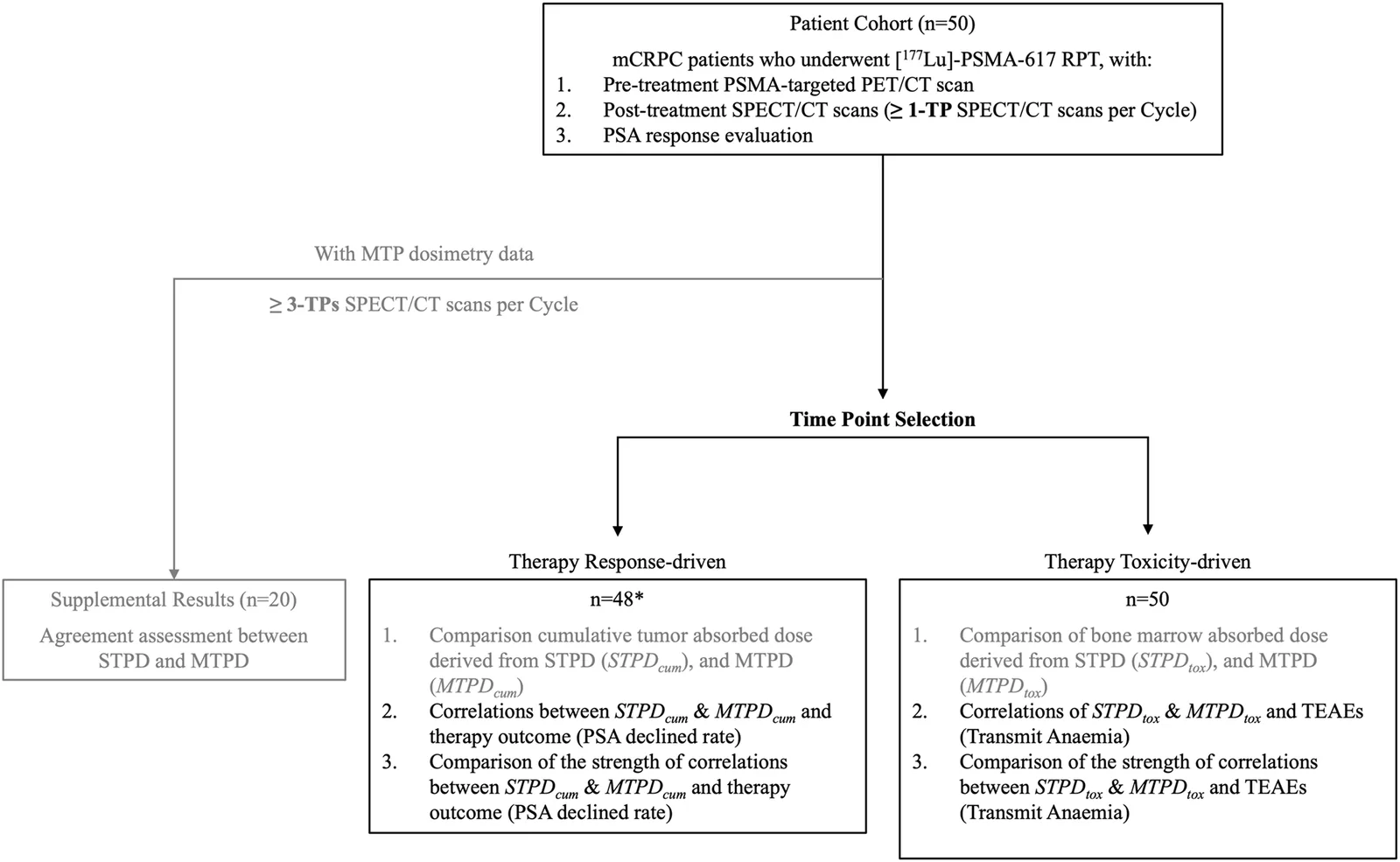
Study Workflow for Outcome-Driven Dosimetry Time-Point Identification Analysis (Dosimetry Comparison and Outcome Correlation) in mCRPC Patients Undergoing [^1^⁷⁷Lu]Lu-PSMA-617 RPT *n=48: Of the 50 patients in the full cohort, 48 had available PSA measurements during therapy. Two of these patients (G2-01, G2-18) lacked first-cycle dosimetry data, precluding calculation of cumulative absorbed dose and direct comparison between *STPD*_*cum*_ and *MTPD*_*cum*_, they were therefore excluded from correlation analyses

The Cantonal Research Ethics Committee review board approved the retrospective, single-center study (Ethical approval numbers: 2023-01877), and all patients provided written informed consent. We collected data from 50 mCRPC patients who received [^177^Lu]Lu-PSMA-617 RPT between 09.2019 and 12.2022 at the Department of Nuclear Medicine, Inselspital, Bern University Hospital. The cohort included 20 patients with MTP dosimetry data, who were previously published in another study [41], and an additional 30 patients who were ineligible for MTPD estimation. All patients underwent at least one post-therapeutic SPECT/CT scan. Pre-therapeutic [^68^Ga]Ga-PSMA-11 PET/CT or [^18^F]F-PSMA-1007 PET/CT was required to confirm PSMA-positive disease. The study workflow was illustrated in Figure 2.

### Dosimetry estimation

#### MTP dosimetry

MTP-based dose-maps (MTPD-maps) were generated using the Hermia Voxel Dosimetry from Hermes Medical Solutions (HERMES, Stockholm, Sweden), utilizing at least 3 acquisitions of SPECT/CT for 20 patients. Briefly, SPECT/CT datasets from different TPs were automatically co-registered to a reference scan, followed by voxel-wise time–activity curve fitting to estimate the time-integrated activity, which was then converted into three-dimensional voxel-wise absorbed dose maps using radionuclide-specific dose kernels for [^177^Lu]Lu (see Supplemental Figure 3).

#### STP dosimetry

STP-based dose-maps (STPD-maps) were generated using a single post-therapeutic SPECT/CT acquisition by VoxelDosimetry’s built-in Hänscheid approximation within the same software framework (see Supplemental Figure 4)[21].

#### Organ-specific dosimetry

OARs, including the kidneys, liver, spleen, and whole-skeletal bone structures, were segmented automatically on SPECT/CT using TotalSegmentator, a deep-learning-based anatomical segmentation framework [42]. Whole-body tumor and bone marrow segmentation was performed semi-automatically using a patient-specific global thresholding approach on pre-therapeutic PET/CT images, adapted from previously described methods [41]: lesion preselection was guided by visual identification of focal uptake, followed by iso-contouring using a threshold relative to the individual patient’s liver uptake [43]. To mitigate partial-volume effects, lesion segmentations were subsequently refined semi-automatically by a second radiologist using LIFEx software, an open-source platform for image-based tumor characterization [44]. Bone marrow was approximated using the whole-skeleton bone volume after exclusion of segmented whole-body tumor regions. The resulting segmentation masks were then automatically registered to post-therapy SPECT/CT and corresponding dose-maps [45, 46]. For MTPD calculations, SPECT-based registration was referenced to the first post-therapeutic acquisition (ca 2-hour post-injection) for spatial alignment. Mean absorbed doses were extracted from all MTPD- and STPD-maps.

### Treatment Outcome-Driven Dosimetry and Optimal Time-point Identification

#### Treatment outcome-driven dosimetry parameter definition

Toxicity evaluation focuses on transient adverse effects, which were correlated with mean absorbed dose from single MTPD- and STPD-maps, referred to *MTPD*_*tox*_ (in gray, Gy), and *1d-STPD*_*tox*_, *2d-STPD*_*tox*_, *3d-STPD*_*tox*_ (in Gy) based on single SPECT acquisitions at 1-, 2-, 3-day(s) p.i., respectively. Cumulative absorbed doses over treatment cycles were computed for 48 patients (2 patients were excluded due to missing the first cycle dosimetry data), referred to as *MTPD*_*cum*_ and *STPD*_*cum*_ (in Gy), respectively.

##### Toxicity-driven optimal time-point identification

Treatment-emergent adverse events (TEAEs) [47], including transient Anaemia (hemoglobin decrease), Leucopenia (white blood cell decreased), and Thrombopenia (Platelet count decreased), were documented and graded according to the Common Terminology Criteria for Adverse Events v5.0 (CTCAE v5.0) [48]. Blood counts were collected 1 day before each treatment cycle, and again 3-4 days after administration (shown in Figure 1). Safety and tolerability represented by the rate of grade ≥3 events were also documented for individuals. *MTPD*_*tox*_ and *STPD*_*tox*_ were correlated with transient TEAEs.

##### Response-driven optimal time-point identification

Treatment response was demonstrated biochemically by serum PSA measurement that was classified according to the recommendations of the Prostate Cancer Working Group 3 (PCWG3) [49]. A good PSA response was defined as a post-therapeutically confirmed PSA decline of ≥50% compared to the baseline PSA value. *MTPD*_*cum*_ and *STPD*_*cum*_ were correlated with the PSA decline rate over cycles.

## Statistical analysis

Frequency analyses, descriptive statistics, statistical comparisons, and correlations were carried out using SPSS software (IBM SPSS Statistics 28.0, New York). Shapiro-Wilk test was used for testing normal distribution. Spearman correlation was used to assess the relationship between dosimetry results (*MTPD*_*cum*_, *MTPD*_*tox*_, *STPD*_*cum*_, and *STPD*_*tox*_) and clinical outcomes, corresponding dose-response relationship was fitted with linear regression. The Mann-Whitney U test was applied to compare dosimetry parameters of different therapy outcome-based subgroups. Correlation analyses were used to explore associations between absorbed dose estimates and clinical outcomes at the cycle level. Repeated observations from the same patient were not modeled as independent, corresponding *p*-values were adjusted for multiple comparisons using the Benjamini-Hochberg false discovery rate (FDR), adjusted *p*-values were marked as *p*_*adj*_. Differences in correlation strength between dosimetry parameters and therapy outcome were compared using Williams’ *F*-test for dependent correlations, accounting for the shared variance. A two-sided *p*-value below 0.05 was considered significant.

## Results

### Patient characteristics: treatment response and toxicity

50 patients (Median age: 70, range: 54-89 years old) received 181 cycles of [^177^Lu]Lu-PSMA-617 RPT. Dosimetry analyses were performed on a per-cycle basis. Depending on imaging availability, some patients contributed data to multiple categories across cycles, cycles contributed MTP dosimetry (≥3 SPECT/CT TPs) or STP dosimetry at 1, 2, or 3 days p.i. (involving 359 TPs of SPECT/CT scans: 1-day p.i.: n = 154, 2-day p.i.: n = 154, 3-day p.i.: n = 51). Mean administered activity was 6.85 GBq (Range: 3.70 - 7.50 GBq), with a 5-8 weeks interval between cycles. No statistically significant differences in baseline characteristics were observed between cycles with MTP and STP dosimetry patients.

PSA responses across 1^st^-6^th^ cycles were depicted in a waterfall plot (Supplemental Figure 1). PSA-decreased responses were found in 38/49 (77.55%) patients after the 1^st^ cycle. Following the 2^nd^ cycle, a good PSA response was observed in 19/39 (48.72%) patients.

Treatment was well tolerated by all patients, with no Grade 4 or higher hematologic, hepatic, or renal toxicity observed. Transient Grade 1 to 3 hematological toxicities, including Anaemia, Leucopenia, and Thrombopenia across cycles, are summarized in Supplemental Table 1.

### Dosimetry estimation & optimal time-point identification in tumor and bone marrow

**Fig. 3:**
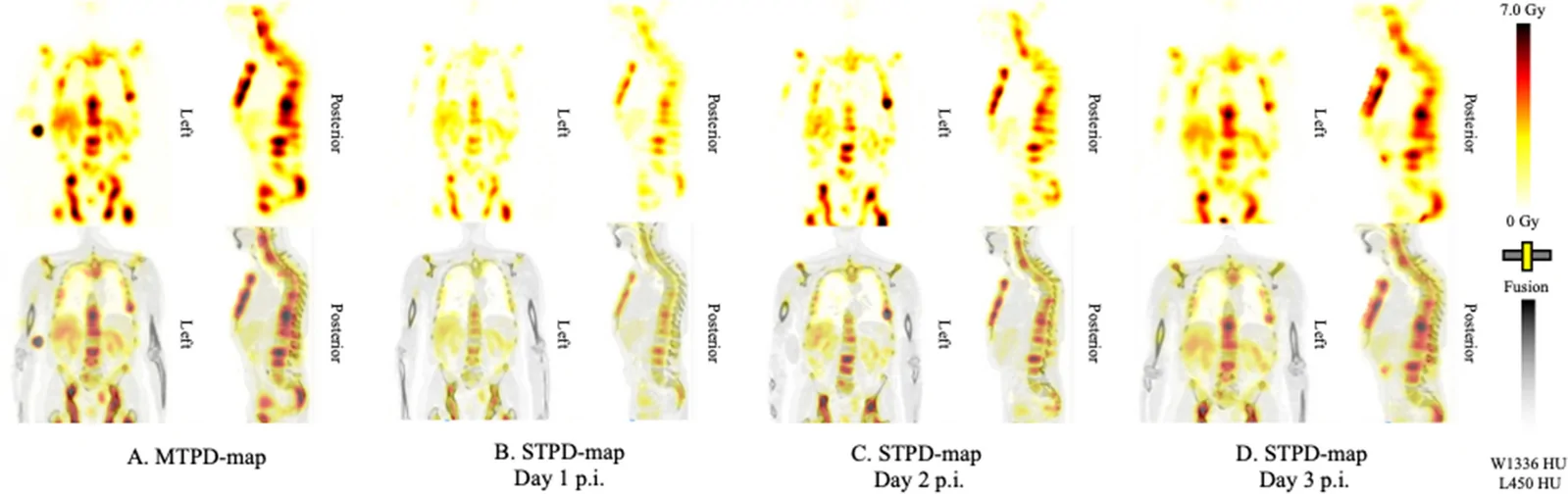
Comparison of STPD-maps on 1-, 2-, and 3-day p.i. to MTPD-map. The fusion view of dose-maps and CT were displayed using identical contrast setting, including the same absolute value range for dose-maps, and consistent window width (W) & window level (L) for the CT images

Representative MTPD-map alongside corresponding STPD-maps are presented in Figure 3. An overview of all available post-injection SPECT/CT imaging time points for individual dosimetry analysis is shown in Supplemental Figure 2. Organ-wise cycle-specific OARs dosimetry (*MTPD*_*tox*_, *1d-STPD*_*tox*_, *2d-STPD*_*tox*_, and *3d-STPD*_*tox*_), and cumulative whole-body tumor dosimetry values (*MTPD*_*cum*_, *1d-STPD*_*cum*_, *2d-STPD*_*cum*_, *3d-STPD*_*cum*_) are summarized in Table 1. Additional normalized dosimetry statistics are documented in Supplemental Table 2.

**Table 1.** Organ-specific *MTPD*_*tox*_ (Gy), and *STPD*_*tox*_ (Gy), and whole-body tumor *MTPD*_*cum*_ (Gy), and *STPD*_*cum*_ (Gy) by cycles

Organ	Mean Dose ± SD (Gy)	1^st^ Cycle	2^nd^ Cycle	3^rd^ Cycle	4^th^ Cycle	5^th^ Cycle	6^th^ Cycle
Bone Marrow	*1d-STPD*_tox_	0.15 ± 0.15 (n=46)	0.16 ± 0.19 (n=34)	0.15 ± 0.19 (n=26)	0.11 ± 0.09 (n=22)	0.11±0.09 (n=16)	0.07±0.04 (n=10)
	*2d-STPD*_tox_	0.41 ± 0.99 (n=42)	0.25 ± 0.36 (n=34)	0.22 ± 0.34 (n=26)	0.16 ± 0.26 (n=25)	0.13±0.14 (n=17)	0.10±0.05 (n=10)
	*3d-STPD*_tox_	0.55 ± 0.69 (n=12)	0.47 ± 0.74 (n=12)	0.34 ± 0.70 (n=9)	0.13 ± 0.07 (n=8)	0.19±0.23 (n=8)	0.11±0.08 (n=2)
	*MTPD*_tox_	0.39 ± 0.34 (n=16)	0.46 ± 0.69 (n=15)	0.40 ± 0.60 (n=13)	0.27 ± 0.32 (n=15)	0.22±0.23 (n=9)	0.16±0.07 (n=6)
Left Kidney	*1d-STPD*_tox_	1.58 ± 0.68 (n=46)	1.77 ± 0.93 (n=34)	1.84 ± 0.89 (n=26)	1.78 ± 0.69 (n=22)	1.85±0.76 (n=16)	1.31±1.08 (n=10)
	*2d-STPD*_tox_	6.49 ± 30.41 (n=42)	2.22 ± 1.43 (n=34)	2.16 ± 1.15 (n=26)	2.19 ± 1.01 (n=25)	1.93±0.93 (n=17)	1.76±1.73 (n=10)
	*3d-STPD*_tox_	2.16 ± 1.28 (n=12)	2.96 ± 1.77 (n=12)	3.07 ± 1.59 (n=9)	2.35 ± 1.98 (n=8)	2.18±1.01 (n=8)	1.83±1.20 (n=2)
	*MTPD*_tox_	2.22 ± 0.92 (n=16)	3.43 ± 1.96 (n=15)	3.03 ± 1.83 (n=13)	2.84 ± 1.26 (n=15)	2.28±0.57 (n=9)	2.01±1.39 (n=6)
Right Kidney	*1d-STPD*_tox_	1.68 ± 0.64 (n=45)	1.98 ± 0.86 (n=34)	2.00 ± 0.90 (n=26)	2.22 ± 0.84 (n=22)	2.14±0.75 (n=16)	1.48±1.12 (n=10)
	*2d-STPD*_tox_	6.80 ± 30.94 (n=41)	2.39 ± 1.32 (n=34)	2.44 ± 1.12 (n=26)	2.72 ± 1.06 (n=25)	2.34±1.07 (n=17)	1.96±1.80 (n=10)
	*3d-STPD*_tox_	2.12 ± 1.01 (n=12)	3.02 ± 1.67 (n=12)	3.23 ± 1.66 (n=9)	2.45 ± 1.50 (n=8)	2.45±1.15 (n=8)	2.08±1.31 (n=2)
	*MTPD*_tox_	2.26 ± 0.96 (n=16)	3.34 ± 1.43 (n=15)	3.02 ± 1.47 (n=13)	3.22 ± 1.09 (n=15)	2.57±0.67 (n=9)	2.40±1.33 (n=6)
Liver	*1d-STPD*_tox_	0.45 ± 0.26 (n=46)	0.48 ± 0.23 (n=34)	0.56 ± 0.24 (n=26)	0.66 ± 0.23 (n=22)	0.60±0.24 (n=16)	0.42±0.25 (n=10)
	*2d-STPD*_tox_	1.37 ± 5.08 (n=42)	0.53 ± 0.29 (n=34)	0.67 ± 0.36 (n=26)	0.67 ± 0.31 (n=25)	0.62±0.30 (n=17)	0.55±0.40 (n=10)
	*3d-STPD*_tox_	0.85 ± 0.64 (n=12)	0.66 ± 0.30 (n=12)	0.69 ± 0.28 (n=9)	0.86 ± 0.41 (n=8)	0.68±0.30 (n=8)	0.55±0.08 (n=2)
	*MTPD*_tox_	0.87 ± 0.58 (n=16)	0.92 ± 0.33 (n=15)	0.83 ± 0.31 (n=13)	0.96 ± 0.36 (n=15)	0.72±0.22 (n=9)	0.59±0.25 (n=6)
Spleen	*1d-STPD*_tox_	0.31 ± 0.16 (n=46)	0.36 ± 0.20 (n=34)	0.42 ± 0.24 (n=26)	0.52 ± 0.27 (n=22)	0.48±0.28 (n=16)	0.33±0.27 (n=10)
	*2d-STPD*_tox_	0.79 ± 2.50 (n=42)	0.38 ± 0.28 (n=34)	0.44 ± 0.29 (n=26)	0.46 ± 0.36 (n=25)	0.49±0.40 (n=17)	0.40±0.39 (n=10)
	*3d-STPD*_tox_	0.55 ± 0.50 (n=12)	0.45 ± 0.30 (n=12)	0.39 ± 0.26 (n=9)	0.56 ± 0.42 (n=8)	0.62±0.52 (n=8)	0.34±0.10 (n=2)
	*MTPD*_tox_	0.57 ± 0.48 (n=16)	0.59 ± 0.20 (n=15)	0.65 ± 0.24 (n=13)	0.80 ± 0.41 (n=15)	0.70±0.47 (n=9)	0.42±0.13 (n=6)
Whole-body Tumor	*1d-STPD*_*cum*_	4.14 ± 3.06 (n=46)	6.92 ± 6.20 (n=34)	8.81 ± 6.40 (n=26)	10.31 ± 7.86 (n=22)	12.03 ± 9.88 (n=16)	10.82 ± 8.02 (n=10)
	*2d-STPD*_*cum*_	13.14 ± 41.52 (n=42)	18.83 ± 46.43 (n=34)	15.83 ± 12.63 (n=26)	15.36 ± 13.32 (n=25)	19.69 ± 16.86 (n=17)	20.51 ± 16.45 (n=10)
	*3d-STPD*_*cum*_	11.04 ± 4.99 (n=12)	15.83 ± 11.61 (n=12)	18.28 ± 18.32 (n=9)	11.04 ± 11.74 (n=8)	24.53 ± 20.47 (n=8)	24.66 ± 4.07 (n=2)
	*MTPD*_*cum*_	7.57 ± 4.76 (n=16)	17.41 ± 13.49 (n=15)	24.73 ± 16.90 (n=13)	22.80 ± 19.28 (n=15)	33.40 ± 20.86 (n=9)	31.22 ± 20.35 (n=6)

#### Toxicity-driven time-point identification: bone marrow dosimetry vs. hematologic

**Fig. 4:**
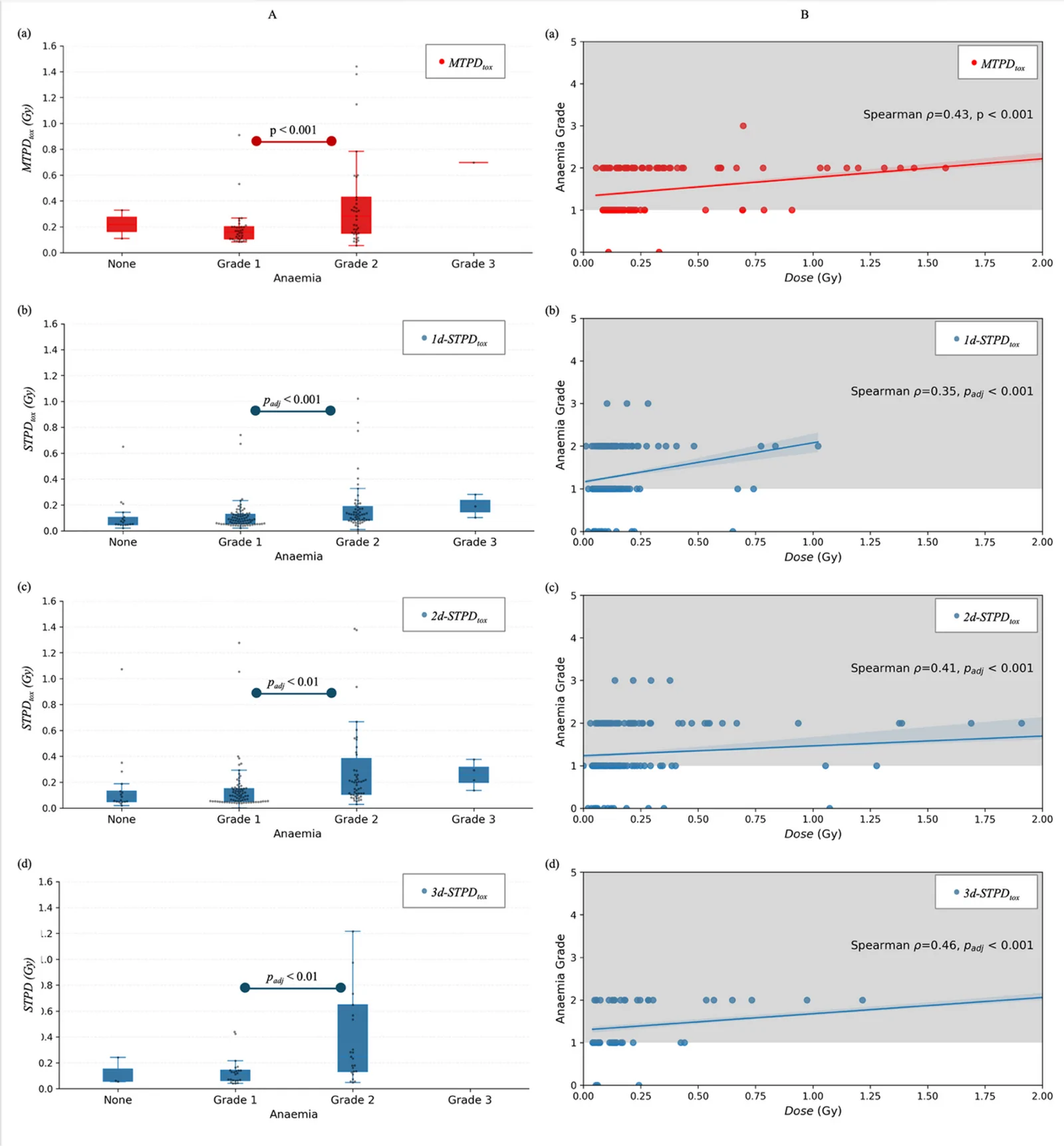
Correlations between Dosimetry Parameters (*STPD*_*tox*_ and *MTPD*_*tox*_) and Therapy Toxicity. A. Box plots of bone marrow dosimetry parameters stratified by Anaemia grades:* MTPD*_*tox*_ (**a**), 1d-*STPD*_*tox*_ (**b**), 2d-*STPD*_*tox*_ (**c**), 3d-*STPD*_*tox*_ (**d**), based on single SPECT acquisitions at 1-, 2-, 3-day p.i. respectively

For bone marrow dosimetry, both STPD- and MTPD- drived absorbed dose showed positive correlations with Anaemia grade 1 and 2 across all evaluated TPs (*1d-*, *2d-*, *3d-STPD*_*tox*_, *p*_*adj*_<0.01), and *MTPD*_*tox*_ (*p*<0.001), as illustrated in Figure 4. Spearman correlation analysis showed that *STPD*_*tox*_ (*1d-STPD*_*tox*_: Spearman’s ρ=0.35, *p*_*adj*_<0.001; *2d-STPD*_*tox*_: Spearman’s ρ=0.41, *p*_*adj*_<0.001; *3d-STPD*_*tox*_: Spearman’s ρ=0.46, *p*_*adj*_<0.001) and *MTPD*_*tox*_ (Spearman’s ρ=0.43, *p*<0.001) demonstrated significant correlations with anemia grades.

Comparing correlations across methods, the inter-method correlation between *STPD*_*tox*_ and *MTPD*_*tox*_, was highest at *1d-* (r=0.79), moderate at *2d-* (r=0.59) and *3d-* (r=0.61). Williams’ test revealed no statistically significant difference between *STPD*_*tox*_ and *MTPD*_*tox*_ derived correlations (*1d-:* F(1,66)=0.38, *p*=0.54; *2d-*: F(1,67)=1.50, *p*= 0.22; *3d-*: F(1,38)=0.71, *p*=0.41).

#### Response-driven time-point identification: tumor dosimetry vs. PSA response

**Fig. 5:**
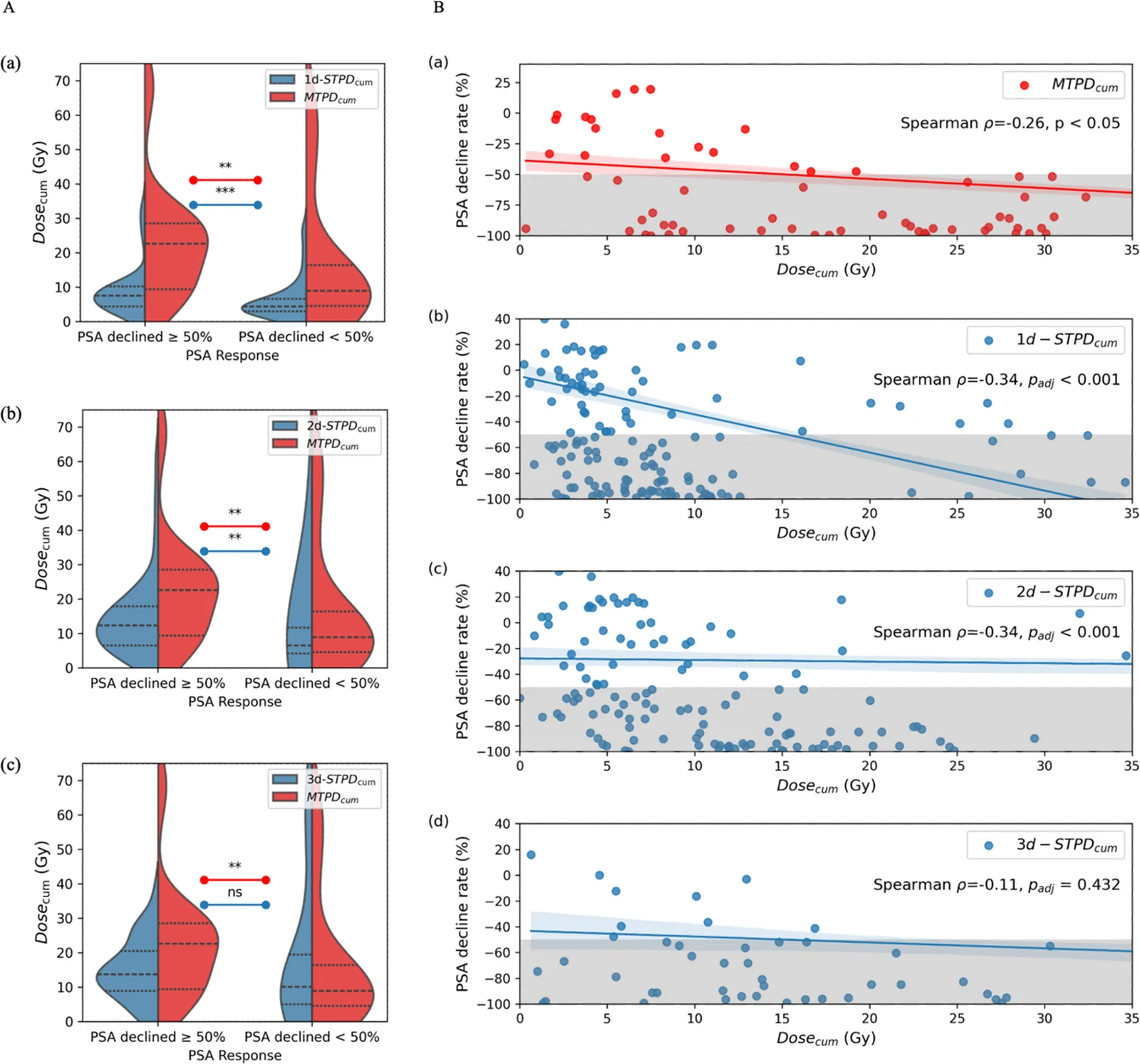
Correlations between Dosimetry Parameters *(MTPD*_*cum*_ and *STPD*_*cum*_*)* and Therapy Response. A. Split violin plots of whole-body tumor *MTPD*_*cum*_, 1d-*STPD*_*cum*_ (**a**), 2d-*STPD*_*cum*_ (**b**), and 3d-*STPD*_*cum*_ (**c**), stratified by PSA decline rate. Significance levels: ns: non-significant, *p(p*_*adj)*_<0.05: *, *p(p*_*adj)*_<0.01: **, *p(p*_*adj*_*)*<0.001: ***. B. Scatter plots with linear regression lines illustrating the relationship between PSA decline rate and whole-body tumor *MTPD*_*cum*_ (**a**), 1d-*STPD*_*cum*_ (**b**), 2d-*STPD*_*cum*_ (**c**), and 3d-*STPD*_*cum*_ (**d**)

For tumor dosimetry, *STPD*_*cum*_ (*1d-STPD*_*cum*_: *p*_*adj*_<0.001, *2d-STPD*_*cum*_: *p*_*adj*_<0.001, *3d-STPD*_*cum*_: *ns*) and *MTPD*_*cum*_ (*p*<0.01) demonstrated significant differences between subgroups with PSA declined 50% and <50% according to U-test (Figure 5.A). Both *STPD*_*cum*_ and *MTPD*_*cum*_ showed modest negative correlations with PSA decline rate (Figure 5.B): *1d-STPD*_*cum*_ (Spearman’s ρ=-0.34, *p*_*adj*_<0.05) and *2d-STPD*_*cum*_ (Spearman’s ρ=-0.34, *p*_*adj*_<0.001), as well as *MTPD*_*cum*_ (Spearman’s ρ=-0.26, *p*<0.001).

The inter-method correlations between *MTPD*_*cum*_ and *STPD*_*cum*_ were modest at all TPs (*1d-*: r= 0.56; *2d-*: r=0.51; *3d-*: r=0.50). Despite these numerical differences, Williams’ test showed no statistically significant difference in correlation strength between *MTPD*_*cum*_ and *STPD*_*cum*_ at any TPs (*1d-*: F(1,66)=0.73, *p*=0.40; *2d-*: F(1,67)=0.00, *p*=0.97; *1d-*: F(1,38)=0.01, *p*=0.92).

## Discussion

This work engages with a longstanding discussion in measurement theory regarding whether measurements should be interpreted primarily as statements about an objective property of the system under study or as operational constructs whose value lies in their effectiveness for decision-making [50]. In RPT, dosimetry can be viewed through two lenses: as an estimation of an underlying physical quantity-absorbed dose-that exists independently of the observer, and as an operational measurement whose clinical value is ultimately determined by its ability to inform patient management. While absorbed dose is firmly grounded in radiation physics, its translation into clinical practice is constrained by practical burdens, including imaging limitations, workflow feasibility, and uncertainties in modeling assumptions. These constraints create a fundamental tension in RPT: fixed-activity administration protocols offer procedural streamlining but neglect patient-specific variability in pharmacokinetics, absorbed dose distribution and biological response. Conversely, fully individualized MTP dosimetry, though theoretically optimal from a physics standpoint, imposes substantial logistical and resource burdens that hinder routine clinical adoption. Against this background, we adopt a pragmatic, outcome-driven perspective, not to redefine absorbed dose as a subjective quantity, but to evaluate whether simplified dosimetric approximations retain clinical relevance under real-world constraints. In this proof-of-concept study, outcome-driven dosimetry is positioned as a complementary evaluation framework, rather than a replacement for physics-driven efforts toward accuracy optimization. Building on our prior work demonstrating the feasibility and clinical relevance of STP dosimetry approaches in [^1^⁷⁷Lu]Lu-PSMA-617 therapy [41], the present study extended dataset by including data from 30 patients ineligible for MTP dosimetry estimation (total n=50), thereby allowing a more comprehensive assessment of outcome-driven dosimetry optimization for optimal TP identification.

Both *STPD*_*tox*_ and *MTPD*_*tox*_ showed positive correlations with anemia grades within 3 days p.i., with the highest correlation observed at 3 days p.i. (Spearman’s ρ=0.46, *p*_*adj*_<0.001). Across all evaluated TPs, inter-method agreement between *MTPD*_*tox*_ and *STPD*_*tox*_ was moderate across TPs (r=0.59–0.79), the strength of correlations with anemia was statistically comparable between methods, and no significant inter-method differences were detected by Williams’ test. For therapeutic response correlation, cumulative doses were evaluated for whole-body tumors, as PSA decline reflects the integrated treatment effect across all irradiated tumor sites and therapy cycles. *MTPD*_*cum*_ and *STPD*_*cum*_ showed moderate inter-method correlations (r=0.50–0.56) and comparable associations with PSA decline rate, with negative correlations observed for both methods (*MTPD*_*cum*_: Spearman’s ρ=-0.26, *p*_*adj*_<0.05; *1d-, 2d-STPD*_*cum*_: Spearman’s ρ=-0.34, *p*_*adj*_<0.001). Taken together, these findings suggested that STP dosimetry within 2-day p.i. may represent a balanced compromise, capture both toxicity- and efficacy-related dose-response information while preserving clinical feasibility without requiring additional workloads. This aligns with previous studies that generally characterized normal organs dosimetry best within the first 2-day p.i. [30, 51, 52]. In contrast, tumor dosimetry tends to benefit in accuracy from later scans beyond 3-day [5, 51]. For example, Hänscheid approximation on 2-day p.i. reported the smallest percentage differences (PD) for kidneys (-1.3±5.6%) [23], and the lowest error at 60 hours (10.18±8.71%) [52]. Regarding tumor dosimetry, Brosch-Lenz et al. reported Hänscheid approximation on 3-day p.i. showed the smallest PD of -1.9±14.8% for isolated tumors [23]. Chen et al. found that at 200 hours resulted in the smallest mean absolute errors (14.69±11.85%) compared to 4-TPs dosimetry [52]. Importantly, these previous studies primarily focused on promoting TPs to minimize dosimetric error relative to MTP reference models, whereas the present analysis prioritizes maximizing correlation with clinical outcomes, which may favor different imaging windows.

Limitations include retrospective nature and small cohort, reduced sample sizes at later cycles, and confounding related to systematic uncertainties in segmentation method, intra-patient correlations and cycle-specific clinical factors cannot be entirely excluded. Additionally, five patients received approximately half of the mean administered activity at their final cycle (four at cycle 6 and one at cycle 4), which may have increased variability and influenced cycle-specific correlations. Potential prior therapies, evolving tumor microenvironment heterogeneity [43, 53–55], and reliance on population-based kinetics may affect accuracy [19], positioning STP as a pragmatic but less precise alternative for biological response estimates. Absorbed dose-toxicity correlation analyses were restricted to hematologic endpoints in this study, longer-term follow-up in larger cohorts will be necessary to evaluate potential associations between absorbed dose and late renal or hepatic toxicity. Observed dose–outcome correlations were modest and should be interpreted as exploratory associations rather than evidence of strong clinical prediction. Generalizability across institutions, imaging protocols, and radiopharmaceuticals requires further validation.

By emphasizing the link between dosimetry and clinical outcomes, this work offers a practical solution to reduce the burden of clinical dosimetry without sacrificing outcome relevance, with Hänscheid approximation within 2-day p.i. as an outcome-driven, physics-supported compromise. Our finding shows potential for direct integration into clinical treatment planning, therefore informing treatment decisions that balance tumor control with healthy tissue preservation. Nevertheless, broader validation across different simplified approaches and diverse patient cohorts remains needed [5, 20, 23, 30, 56]. This supports a shift toward outcome-oriented strategies that prioritize theragnostic value over exhaustive physical accuracy-driven sampling.

## Conclusion

Beyond methodological optimization, this work engages with a longstanding debate in measurement theory by framing dosimetry not only as an estimate of an underlying physical quantity, but also as an operational tool whose value is ultimately defined by its clinical utility. From the proof-of-concept, outcome-driven perspective, by linking bone marrow dosimetry to hematologic toxicity and tumor dosimetry to PSA decline rate, we demonstrated that the Hänscheid approximation within 2-day p.i. emerges as a practical clinical compromise, balancing physics-based accuracy with clinical relevance. Integrating the proposed outcome-driven dosimetry strategies into routine practice could advance personalized dosimetry and facilitate broader implementation in radiopharmaceutical therapy.

## Supplementary Information

Below is the link to the electronic supplementary material.Supplementary file1 (PNG 290 KB)Supplementary file2 (PNG 588 KB)Supplementary file3 (PNG 469 KB)Supplementary file4 (PNG 948 KB)Supplementary file5 (DOCX 27 KB)

## Data Availability

The datasets generated during and/or analyzed during the current study are available from the corresponding author on reasonable request.

## Abbreviations

MTP: Multiple-time-point
STP: Single-time-point
TPs: Time-points
TEAEs: Transient treatment-emergent adverse events
RPT: Radiopharmaceutical therapy
mCRPC: Metastatic castration-resistant prostate cancer
MTPD-maps: MTP-based dose-maps
STPD-maps: STP-based dose-maps
CTCAE v5.0: Common Terminology Criteria for Adverse Events
