# Supplementary material for: Outcome-driven dosimetry optimization for [177Lu]Lu-PSMA-617 radiopharmaceutical therapy: proof of concept on single time point dosimetry optimization
Source: Eur J Nucl Med Mol Imaging. 2026 Jun 13;53(11):6461–72. doi: 10.1007/s00259-026-07936-w (PMC13525968; doi:10.1007/s00259-026-07936-w)
Supplement: Supplementary file 1 [file 259_2026_7936_MOESM5_ESM.docx]

## Supplemental Table 1.

| *TEAEs Grading* | | 1^st^ Cycle | 2^nd^ Cycle | 3^rd^ Cycle | 4^th^ Cycle | 5^th^ Cycle | 6^th^ Cycle |
| --- | --- | --- | --- | --- | --- | --- | --- |
| *Anaemia* | *None* | 14.58% (7/48) | 10.53% (4/38) | 10.71% (3/28) | 7.41% (2/27) | 5.56% (1/18) | 10.00% (1/10) |
|  | *Grade 1* | 37.50% (18/48) | 50.00% (19/38) | 60.71% (17/28) | 62.96% (17/27) | 66.67% (12/18) | 40.00% (4/10) |
|  | *Grade 2* | 41.67% (20/48) | 36.84% (14/38) | 28.57% (8/28) | 29.63% (8/27) | 27.78% (5/18) | 50.00% (5/10) |
|  | *Grade 3* | 6.25% (3/48) | 2.63% (1/38) | 0 (0/28) | 0 (0/27) | 0 (0/18) | 0 (0/10) |
| *Leucopenia* | *None* | 100.00% (48/48) | 97.37% (37/38) | 92.86% (26/28) | 88.89% (24/27) | 100.00% (18/18) | 100.00% (10/10) |
|  | *Grade 1* | 0 (0/48) | 0 (0/38) | 7.14% (2/28) | 11.11% (3/27) | 0 (0/18) | 0 (0/10) |
|  | *Grade 2* | 0 (0/48) | 0 (0/38) | 0 (0/28) | 0 (0/27) | 0 (0/18) | 0 (0/10) |
|  | *Grade 3* | 0 (0/48) | 2.63% (1/38) | 0 (0/28) | 0 (0/27) | 0 (0/18) | 0 (0/10) |
| *Thrombopenia* | *None* | 81.25% (39/48) | 68.42% (26/38) | 71.43% (20/28) | 66.67% (18/27) | 83.33% (15/18) | 80.00% (8/10) |
|  | *Grade 1* | 10.42% (5/48) | 23.68% (9/38) | 21.43% (6/28) | 29.63% (8/27) | 5.56% (1/18) | 20.00% (2/10) |
|  | *Grade 2* | 8.33% (4/48) | 7.89% (3/38) | 7.14% (2/28) | 0 (0/27) | 0 (0/18) | 0 (0/10) |
|  | *Grade 3* | 0 (0/48) | 0 (0/38) | 0 (0/28) | 3.70% (1/27) | 11.11% (2/18) | 0 (0/10) |

### ***Supplemental Table 1.*** *Individual TEAEs counts for transient haematotoxicities (None, and Grade 1 to Grade 3) across cycles are summarized for the entire cohort.*

## Supplemental Table 2.

| Organ | Doses  Per Unit Activity (Gy/GBq) | 1^st^ Cycle | 2^nd^ Cycle | 3^rd^ Cycle | 4^th^ Cycle | 5^th^ Cycle | 6^th^ Cycle |
| --- | --- | --- | --- | --- | --- | --- | --- |
| Bone Marrow | 1d-STPD* | 0.02 ± 0.02 (n=46) | 0.02 ± 0.03 (n=34) | 0.02 ± 0.03 (n=26) | 0.02 ± 0.02 (n=22) | 0.02 ± 0.01 (n=16) | 0.01 ± 0.01 (n=10) |
|  | 2d-STPD* | 0.06 ± 0.15 (n=42) | 0.04 ± 0.06 (n=34) | 0.03 ± 0.05 (n=26) | 0.02 ± 0.04 (n=25) | 0.02 ± 0.02 (n=17) | 0.02 ± 0.01 (n=10) |
|  | 3d-STPD* | 0.08 ± 0.10 (n=12) | 0.07 ± 0.12 (n=12) | 0.05 ± 0.10 (n=9) | 0.02 ± 0.01 (n=8) | 0.03 ± 0.04 (n=8) | 0.02 ± 0.01 (n=2) |
|  | MTPD* | 0.06 ± 0.05 (n=16) | 0.07 ± 0.11 (n=15) | 0.06 ± 0.09 (n=13) | 0.04 ± 0.05 (n=15) | 0.03 ± 0.04 (n=9) | 0.03 ± 0.02 (n=6) |
| Left Kidney | 1d-STPD* | 0.22 ± 0.10 (n=46) | 0.25 ± 0.13 (n=34) | 0.26 ± 0.13 (n=26) | 0.26 ± 0.10 (n=22) | 0.28 ± 0.12 (n=16) | 0.27 ± 0.18 (n=10) |
|  | 2d-STPD* | 0.95 ± 4.47 (n=42) | 0.32 ± 0.22 (n=34) | 0.30 ± 0.17 (n=26) | 0.32 ± 0.16 (n=25) | 0.29 ± 0.15 (n=17) | 0.35 ± 0.27 (n=10) |
|  | 3d-STPD* | 0.30 ± 0.17 (n=12) | 0.44 ± 0.27 (n=12) | 0.45 ± 0.23 (n=9) | 0.36 ± 0.28 (n=8) | 0.33 ± 0.16 (n=8) | 0.31 ± 0.20 (n=2) |
|  | MTPD* | 0.31 ± 0.13 (n=16) | 0.50 ± 0.31 (n=15) | 0.43 ± 0.26 (n=13) | 0.42 ± 0.17 (n=15) | 0.34 ± 0.10 (n=9) | 0.37 ± 0.20 (n=6) |
| Right Kidney | 1d-STPD* | 0.24 ± 0.09 (n=45) | 0.28 ± 0.12 (n=34) | 0.28 ± 0.13 (n=26) | 0.32 ± 0.11 (n=22) | 0.32 ± 0.12 (n=16) | 0.32 ± 0.20 (n=10) |
|  | 2d-STPD* | 0.99 ± 4.55 (n=41) | 0.35 ± 0.20 (n=34) | 0.34 ± 0.16 (n=26) | 0.40 ± 0.16 (n=25) | 0.35 ± 0.17 (n=17) | 0.40 ± 0.29 (n=10) |
|  | 3d-STPD* | 0.29 ± 0.14 (n=12) | 0.45 ± 0.25 (n=12) | 0.47 ± 0.24 (n=9) | 0.38 ± 0.22 (n=8) | 0.37 ± 0.19 (n=8) | 0.35 ± 0.22 (n=2) |
|  | MTPD* | 0.31 ± 0.13 (n=16) | 0.49 ± 0.23 (n=15) | 0.43 ± 0.21 (n=13) | 0.48 ± 0.15 (n=15) | 0.39 ± 0.13 (n=9) | 0.45 ± 0.19 (n=6) |
| Liver | 1d-STPD* | 0.06 ± 0.04 (n=46) | 0.07 ± 0.03 (n=34) | 0.08 ± 0.03 (n=26) | 0.10 ± 0.05 (n=22) | 0.09 ± 0.03 (n=16) | 0.09 ± 0.04 (n=10) |
|  | 2d-STPD* | 0.20 ± 0.75 (n=42) | 0.08 ± 0.04 (n=34) | 0.09 ± 0.05 (n=26) | 0.10 ± 0.07 (n=25) | 0.09 ± 0.04 (n=17) | 0.11 ± 0.06 (n=10) |
|  | 3d-STPD* | 0.12 ± 0.09 (n=12) | 0.10 ± 0.04 (n=12) | 0.10 ± 0.04 (n=9) | 0.15 ± 0.11 (n=8) | 0.10 ± 0.04 (n=8) | 0.09 ± 0.01 (n=2) |
|  | MTPD* | 0.12 ± 0.08 (n=16) | 0.13 ± 0.05 (n=15) | 0.12 ± 0.04 (n=13) | 0.15 ± 0.10 (n=15) | 0.11 ± 0.03 (n=9) | 0.11 ± 0.04 (n=6) |
| Spleen | 1d-STPD* | 0.04 ± 0.02 (n=46) | 0.05 ± 0.03 (n=34) | 0.06 ± 0.03 (n=26) | 0.08 ± 0.04 (n=22) | 0.07 ± 0.04 (n=16) | 0.07 ± 0.04 (n=10) |
|  | 2d-STPD* | 0.11 ± 0.37 (n=42) | 0.06 ± 0.04 (n=34) | 0.06 ± 0.04 (n=26) | 0.07 ± 0.05 (n=25) | 0.07 ± 0.06 (n=17) | 0.08 ± 0.05 (n=10) |
|  | 3d-STPD* | 0.08 ± 0.07 (n=12) | 0.07 ± 0.04 (n=12) | 0.06 ± 0.04 (n=9) | 0.09 ± 0.06 (n=8) | 0.09 ± 0.07 (n=8) | 0.06 ± 0.02 (n=2) |
|  | MTPD* | 0.08 ± 0.07 (n=16) | 0.09 ± 0.03 (n=15) | 0.09 ± 0.04 (n=13) | 0.12 ± 0.06 (n=15) | 0.10 ± 0.07 (n=9) | 0.08 ± 0.02 (n=6) |
| Tumor | 1d-STPD* | 0.59 ± 0.43 (n=46) | 0.42 ± 0.41 (n=34) | 0.23 ± 0.22 (n=26) | 0.17 ± 0.14 (n=22) | 0.16 ± 0.15 (n=16) | 0.11 ± 0.10 (n=10) |
|  | 2d-STPD* | 1.89 ± 6.11 (n=42) | 0.70 ± 0.79 (n=34) | 0.41 ± 0.42 (n=26) | 0.27 ± 0.33 (n=25) | 0.23 ± 0.28 (n=17) | 0.22 ± 0.20 (n=10) |
|  | 3d-STPD* | 1.51 ± 0.67 (n=12) | 1.07 ± 0.92 (n=12) | 0.50 ± 0.77 (n=9) | 0.23 ± 0.11 (n=8) | 0.34 ± 0.40 (n=8) | 0.17 ± 0.16 (n=2) |
|  | MTPD* | 1.06 ± 0.69 (n=16) | 0.77 ± 1.03 (n=15) | 0.36 ± 0.67 (n=13) | 0.28 ± 0.28 (n=15) | 0.20 ± 0.28 (n=9) | 0.22 ± 0.16 (n=6) |

### ***Supplemental Table 2. Doses (MTPD* & STPD*) per unit activity:*** *Organ-specific MTPD*, and STPD* by cycles. Mean ± SD values are given.*

** MTPD and STPD refer to the mean absorbed per unit activity extracted from MTPD- and STPD-maps.*
